# miRNA-29a inhibits atherosclerotic plaque formation by mediating macrophage autophagy via PI3K/AKT/mTOR pathway

**DOI:** 10.18632/aging.203951

**Published:** 2022-03-14

**Authors:** Weihua Shao, Suxing Wang, Xiaoxi Wang, Lixia Yao, Xiaoye Yuan, Dai Huang, Bonan Lv, Yuquan Ye, Hongyuan Xue

**Affiliations:** 1 Second Department of Geriatrics, Hebei Medical University and Hebei General Hospital, Shijiazhuang 050051, Hebei, China; 2 Second Department of Geriatrics, Hebei General Hospital, Shijiazhuang 050051, Hebei, China; 3 Medical Examination Center, Hebei General Hospital, Shijiazhuang 050051, Hebei, China; 4 Ultrasound Department, Hebei General Hospital, Shijiazhuang 050051, Hebei, China; 5 Vascular Surgery, Hebei General Hospital, Shijiazhuang 050051, Hebei, China; 6 Ultrasound Department, Hebei Medical University and Hebei General Hospital, Shijiazhuang 050051, Hebei, China

**Keywords:** PI3K/AKT/mTOR, miR-29a, macrophage, plaque, atherosclerotic, atherosclerosis

## Abstract

Background: miR-29a plays a vital role in AS, but the relationship between the miR-29a-targeted PI3K signaling pathway and AS remains unclear. Therefore, this study was carried out.

Methods: Gene expression profiles from the GEO database containing AS samples were analyzed. ApoE^−/−^ mice and RAW264.7 cells were treated with miR-29a negative control (NC), miR-29a mimic and miR-29a inhibitor to establish the AS model. Then MOVAT staining, TEM, Western blotting, and immunofluorescence staining were adopted for testing target proteins.

Results: DEGs were identified from GSE137578, GSE132651, GSE113969, GSE43292, and GSE97210 datasets. It was found that there were targeted binding sites between miR-29a and PIK3CA. Besides, GO and KEGG analysis demonstrated that autophagy was an enriched pathway in AS. Later, PPI network was depicted, and hub genes were then determined. The results revealed that miR-29a suppressed the areas of plaques and lesional macrophages, but had no impact on VSMCs. TEM results showed the organelles pyknosis of lesional macrophages damaged morphological changes. Furthermore, miR-29a amplified the M2-like macrophages but suppressed the polarization of M1-like macrophages in atherosclerotic plaques. According to mouse and RAW 264.7 cell experiments, miR-29a significantly inhibited the protein expressions of PI3K, p-PI3K, p-AKT, and p-mTOR, which were consistent with the increased expressions of autophagy-related proteins, Beclin 1 and LC3II. However, the miR-29a suppression exhibited the contrary results.

Conclusion: MiR-29a elevation induces the increase of autophagy by down-regulating the PI3K/AKT/mTOR pathway in the progression of AS, indicating that miR-29a is a novel therapeutic strategy for AS.

## INTRODUCTION

Cardiovascular and cerebrovascular diseases are the leading causes of death and disability in most industrialized countries, and they are increasingly prevalent in developing countries [1]. Although the diagnosis and treatment of atherosclerotic cardiovascular disease (ASCVD) have been substantially improved in recent decades, its incidence and mortality rates remain the highest [2]. Atherosclerosis (AS) is the basis of ASCVD onset, mainly manifested by vascular lesions and ischemia of the affected organs [3]. Currently, it is considered that lipid metabolism disorder, oxidative stress, and inflammatory response are essential factors for the occurrence and development of AS [4]. A study of Onorati et al. [5] showed that oxidative stress promoted autophagy in plaque cells under the phosphoinositide 3-kinase/protein kinase B/mammalian target of rapamycin (PI3K/AKT/mTOR) signaling pathway. Further, one study revealed that the Bushen Kangshuai tablet could effectively restrain AS formation via inhibiting the PI3K/AKT/mTOR signaling pathway [6]. Despite optimal treatment with modern interventions and drugs, the recurrence rate of AS is still very high in the first 12 months, about 10% [7]. Therefore, the transcriptomic analysis on the genetic susceptibility to diseases should be furthermore studied, which can provide a complete explanation of the pathogenesis of AS and provide new biomarkers and therapeutic strategies for AS.

Micro ribonucleic acids (miRNAs) are single-stranded RNAs of 22-nucleotides in length that play a critical role in regulating gene expression in multi-cellular organisms. Recently, many studies have reported the inseparable relationship between miRNAs and AS, which are involved in the functional regulation of mononuclear macrophages [8–10]. In addition, Jian et al. have confirmed that miR-29a could promote the secretion of pro-inflammation factors such as interleukin-1 (IL-1), IL-6, and TNF-α and affect the formation of atherosclerotic plaques by regulating the survival status of macrophages [11]. However, the underlying molecular mechanisms of miR-29a in AS are not fully clear. In the present study, we investigated whether miR-29a could enhance the stability of atherosclerotic plaques and inhibit the AS progression-activated macrophage autophagy by down-regulating the PI3K/AKT/mTOR signaling pathway.

## MATERIALS AND METHODS

### Microarray expression profiling and differential expression analysis

GSE137578, GSE132651, GSE113969, GSE43292 and GSE97210 datasets were downloaded from Gene Expression Omnibus (GEO). The edgeR package [12] was employed to convert the original microarray data into expression measures. The differentially expressed genes (DEGs) were analyzed by intersecting function in the Limma R package [13]. Subsequently, PicTar, TargetScan, starBase, and mirDIP were used for predicting miR-29a targets. Finally, Gene Ontology (GO) functional annotation and Kyoto Encyclopedia of Genes and Genomes (KEGG) pathway analysis were performed using Gene-Sifter software and DAVID online tool.

### Model establishment

A total of 30 male ApoE^−/−^ mice (aged 6–8 weeks old) were purchased from Changzhou Cavens Experimental Animal Co., Ltd (Changzhou, China). These mice were fed with a high-cholesterol (1.25%) diet for 4 weeks to establish an early AS model. When AS was measurable, mice were randomly divided into three groups (*n* = 10 in each group): model group [negative control (NC)], miR-29a group (intravenous injection of 1 × 10^9^ pfu lentiviruses containing miR-29a plasmid), and anti-miR-29a group (intravenous injection of 1 × 10^9^ pfu lentiviruses containing anti-miR-29a plasmid). The mice were fed for 4 consecutive weeks and killed immediately after they were intraperitoneally anesthetized with 1% sodium pentobarbital. Then, after perfusion with icy normal saline, the aortic root was extracted from the left ventricle for further experiment. The study was performed in accordance with the *Guide for the Care and Use of Laboratory Animals* of the National Institutes of Health and approved by the Animal Ethics Committees of Hebei General Hospital (Approval No.201904333).

### Histology of lesions and histological analysis

The aortic roots were used for histological evaluation. Specifically, tissues were sectioned and stained correspondingly for each marker. Then 3 cross-sections were quantified for each mouse, with 10 mice per group in all experiments. The computerized image analysis software, Image Pro Plus 6.0, was used for morphological evaluation, and the areas of atherosclerotic plaques, lesional macrophages and vascular small muscle cells (VSMCs) at the aortic roots were measured directly, and the positive areas in atherosclerotic plaques in each mouse were collected and calculated for statistical analysis. Moreover, immunohistochemical staining of MAC-3 and alpha-smooth muscle actin (α-SMA) was carried out to detect the markers for macrophages and VSMCs, respectively, in mouse atherosclerotic lesions [14, 15].

### MOVAT pentachrome staining

After aortic root sections were embedded in paraffin, MOVAT staining was performed to assess the size of arterial plaques under a microscope. Briefly, the nucleus was stained by Wiegert’s hematoxylin, the cytoplasm by Woodstain Scarlet-Acid Fuchsin, elastic tissues by Resorcin-Fuchsin, collagen tissues by Saffron and the matrix by Alcian blue, after which the collagen tissues, proteoglycans, muscle fibers, elastic fibers and fibrins were displayed in 5 different colors on one tissue sections [16].

### Immunofluorescence and immunohistochemistry detection

Aortic root tissue samples were fixed by 4% paraformaldehyde and embedded in paraffin, and glass coverslips of RAW264.7 cells were fixed and used for immunofluorescence staining. Then the samples were de-paraffinized, hydrated and blocked with endogenous peroxidase, followed by microwave treatment with 10 mmol/L sodium citrate buffer for 30 min. Next, the sections were incubated with primary antibodies against MAC-3 (macrophage marker, diluted at 1: 50) and α-SMA (VSMC marker, diluted at 1: 100) for immunohistochemical staining. Later, M1-like macrophage markers, IL-1β, iNOS and interferon gamma (IFN-γ), and M2-like macrophage markers, IL-10, Arginase-1 and MRC-1 were subjected to immunofluorescence staining overnight at 4°C. Washed three times with phosphate-buffered saline (PBS), the samples were incubated with fluorescent or HRP-labeled secondary antibody for 1 h. All antibodies were purchased from Abcam (Cambridge, MA, USA). Subsequently, the samples were sealed with an anti-fluorescent quench agent after PBS washing. Cell nuclei were counterstained by exposure to DAPI for 5 min. Afterwards, the images were captured under a fluorescence microscope (DMM-300D, Shanghai Caikon Optical Instrument Co., Ltd., Shanghai, China).

### Cell culture and transfection

The mouse RAW264.7 cells were purchased from American Type Culture Collection (ATCC, USA). They were cultured in Dulbecco’s Modified Eagle’s Medium (DMEM) containing 10% fetal bovine serum (FBS) and 1% streptomycin, and incubated in 5% CO_2_ at 37°C. When the cell growth density reached 80%, RAW264.7 cells were divided into three groups and transfected with NC mimic, miR-29a mimic, and anti-miR-29a mimic (Ribobio, Guangzhou, China), respectively, by the Lipofectamine 2000 transfection reagents (Invitrogen, Carlsbad, CA, USA) according to the instructions. Then, the cells were cultured in a 24-well plate for 24 h for the following experiments.

### Luciferase assay

Using TargetScan database (http://www.targetscan.org), a putative hsa-miR-29a binding site (CAUUCC) was found in the 3′-untranslated regions (3′-UTR) of PI3K gene. Wild type (WT) and mutant (MUT) plasmids, pmirGLO-PI3K-3′-UTR-WT and pmirGLO-PI3K-3′-UTR-MUT, were provided by Shanghai GenePharma Biology Co., Ltd., and deoxyribonucleic acids (DNAs) were derived from human PI3K complementary DNAs (cDNAs). 293T cells were co-transfected with the constructed plasmids and hsa-miR-29a (NC/MIC) and incubated for 48 h. After cell collection, μL of cell lysate was added to fully lyse 500 cells in each group. Next, 20 μL of cell lysate and 100 μL of Firefly luciferase reaction solution were added to a black microplate, and mixed evenly by vibration to detect the fluorescence value of Firefly luciferase. Later, 100 μL of Renilla luciferase reaction solution was added into the microplate and evenly mixed by vibration to detect the fluorescence value of Renilla luciferase, and the fluorescence value ratio between the two was finally calculated. In each group, 3 replicate wells were set in each experiment.

### Transmission electron microscopy (TEM)

The aortic root tissues were fixed in 0.1 mol/L sodium cacodylate-buffered solution (pH 7.4) and 2.5% glutaraldehyde solution overnight at 4°C, then postfixed in 1% buffered osmium tetroxide for 1 h. The samples were cut into 1-μm sections and stored in 2.5% glutaraldehyde for less than one month. After the sections were stained with uranyl acetate and lead citrate, the changes of macrophage status at the AS aortic roots were identified under the JEM-1200 electron microscope (JEOL Ltd., Tokyo, Japan).

### Western blotting

The descending aortas were used for Western blotting assay. In detail, the total protein was extracted using RIPA lysis buffer (Beyotime Institute of Biotechnology, Inc., Haimen, China). Then the protein samples were separated by sodium dodecyl sulfate-polyacrylamide gel electrophoresis (SDS-PAGE) and transferred onto a polyvinylidene difluoride (PVDF) membrane (Millipore, USA). After 2 h of blocking by Tris Buffered Saline with Tween 20 (TBST) buffer with 5% non-fat milk, the PVDF membrane was incubated overnight at 4°C with primary antibodies against phosphorylated (p)-PI3K p85 (Y458) + PI3 Kinase p55 (Y199), p-AKT (Ser473), p-mTOR (Ser2448), PI3K, AKT, mTOR, Beclin 1, P62 and microtubule-associated protein 1A/1B-light chain 3 II (LC3II) (diluted at 1:1000, Abcam or CST). Following that, the membrane was incubated with the secondary antibody for 1 h. The intensity of protein expression was detected by ECL chemiluminescence (PerkinElmer, Inc., Boston, MA, USA), with GAPDH as an internal normalization control.

### Statistical analysis

All the experiments were repeated independently three times. The data were statistically analyzed by SPSS 21 software, and all measurement data were expressed as mean ± standard deviation. One-way ANOVA followed by Dunnett’s multiple comparisons was applied to assess the differences between groups. *P* < 0.05 represented that the difference was statistically significant.

## RESULTS

### Identification and integration of DEGs in AS

**Figure 1:**
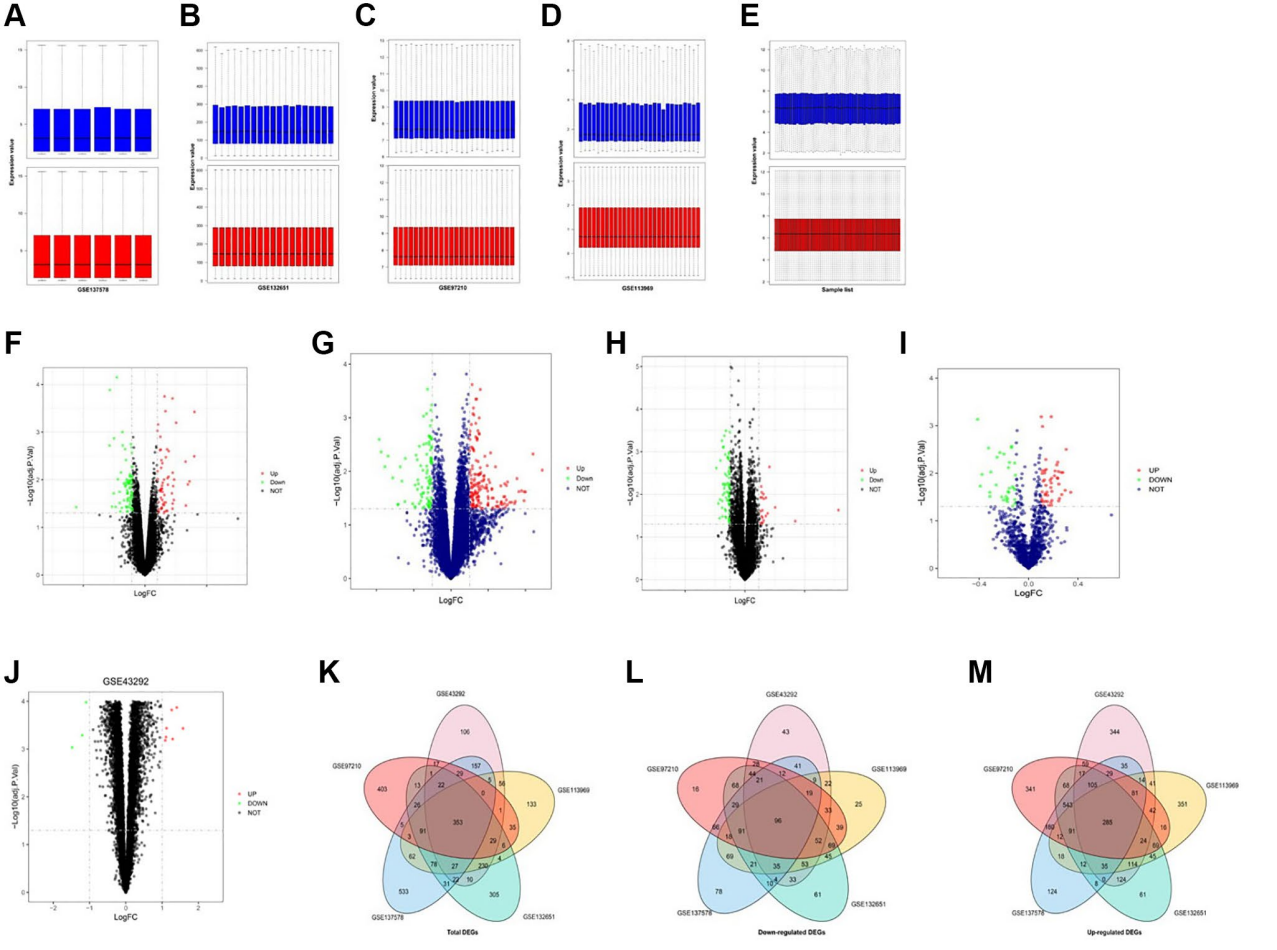
**Identification and integration of DEGs in AS.** (**A**–**E**) Pre-standardization and post-standardization gene expressions of GSE137578, GSE132651, GSE113969, GSE43292 and GSE97210 are demonstrated. (**E**–**H**) DEGs of each dataset are assessed by criteria of *P* < 0.05 and |log2FC|≥2. (**F**–**J**) Volcano maps of the top 100 DEGs of GSE137578, GSE132651, GSE113969, GSE97210 and GSE43292. (**K**–**M**) The total upregulated and downregulated DEGs of GSE137578, GSE132651, GSE113969, GSE43292, and GSE97210 are overlapped and depicted by the Venn diagram analysis.

We analyzed gene expression profiles from the Gene Expression Omnibus (GEO) database GSE137578, GSE132651, GSE113969, GSE43292, and GSE97210, which contained AS samples. Gene expression levels of the 5 GEO datasets were standardized by the quartile division analysis, and the data of pre-standardization and post-standardization were depicted in Figure 1A–1E. The DEGs were assessed by paired-sample *t*-tests within each dataset with criteria of *P* < 0.05 and |log2FC|≥2 (Figure 1E–1H). Later, volcano maps were depicted with the top 100 DEGs of GSE137578 (Figure 1F), GSE132651 (Figure 1G), GSE113969 (Figure 1H), and GSE97210 (Figure 1I), and GSE43292 (Figure 1J). Through the Venn diagram analysis, the integrated DEGs of each dataset were obtained and depicted in Figure 1K–1M.

### GO and KEGG enrichment analyses of DEGs

**Figure 2:**
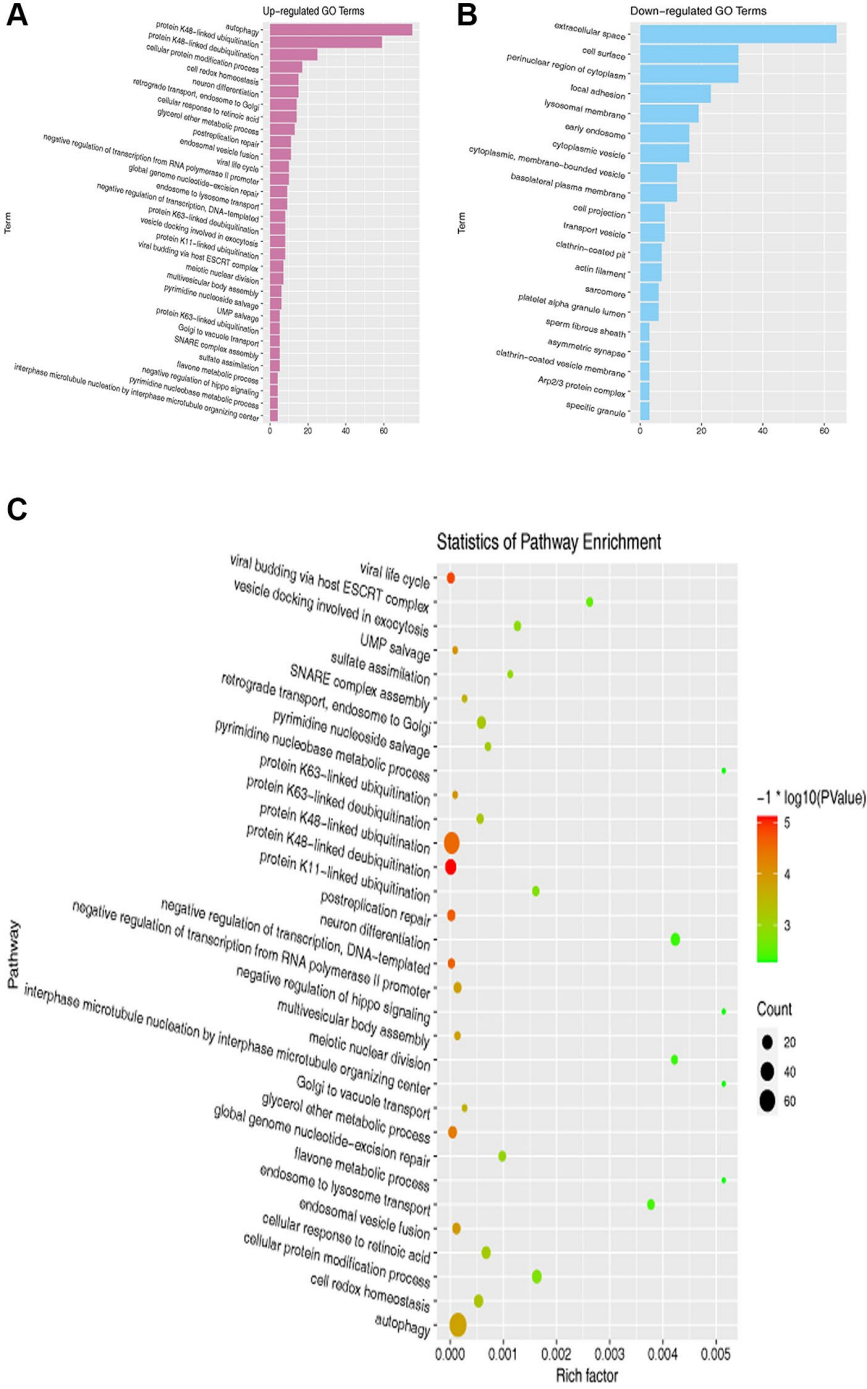
**GO and KEGG pathway enrichment analyses of DEGs.** (**A**, **B**) GO enrichment analysis of overlapping DEGs. (**C**) KEGG pathway enrichment analysis of overlapping DEGs.

Next, we performed GO and KEGG enrichment analyses of the overlapping DEGs among the datasets. The results exposed that DEGs were enriched in the GO pathways, including those related to autophagy, extracellular space, endosomal vesicle fusion, and cell surface (Figure 2A, 2B). In the KEGG enrichment analysis, we found that DEGs were enriched in pathways including those associated with cellular protein modification process, autophagy, and cellular response to retinoic acid (Figure 2C).

### Protein-protein interaction (PPI) network composition and hub gene selection

**Figure 3:**
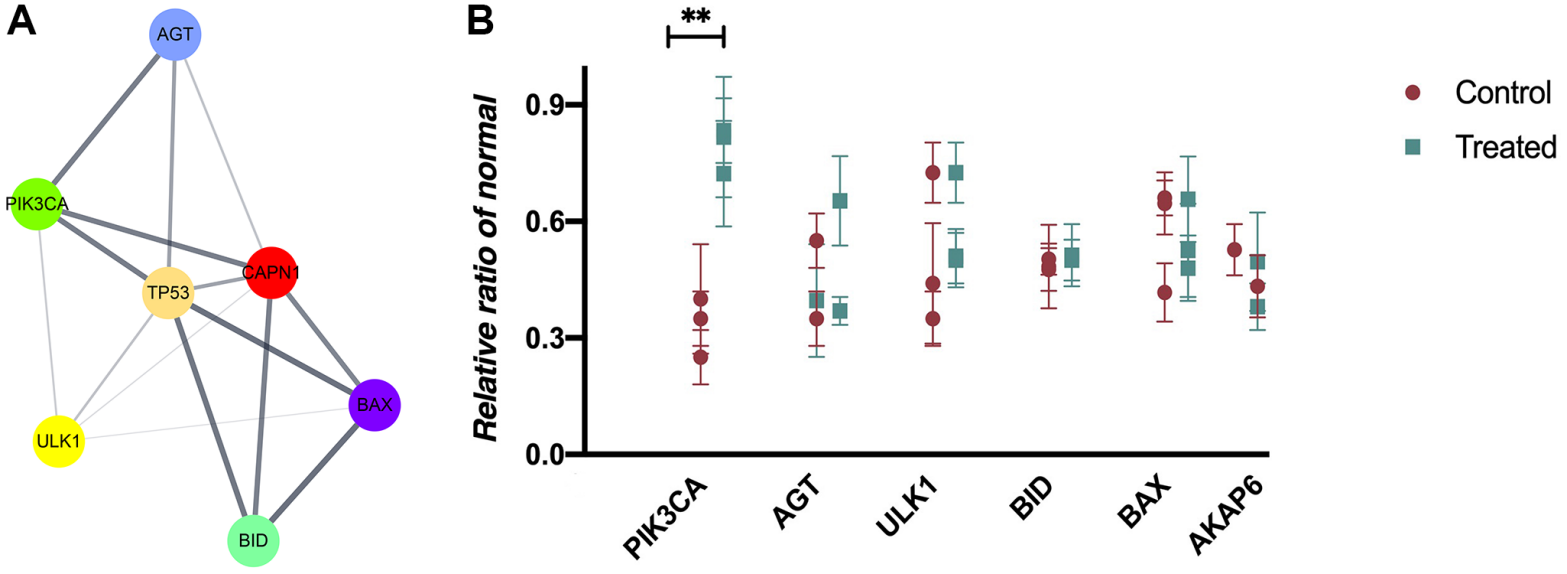
**PPI network composition and hub gene selection.** (**A**) Cytoscape software and STRING database are used to construct a PPI network on the overlapping DEGs. (**B**) The mRNA expressions in mouse blood from control and AS groups (*P* < 0.05).

We constructed the PPI network on the overlapping DEGs using Cytoscape software and STRING database. The results of PPI information were depicted in Figure 3A. 6 genes with the highest scores were obtained as hub genes and depicted in Figure 3B. Compared with those in control group, there was a higher expression of PIK3CA in the AS group (*P* < 0.05, ANOVA), but no statistically significant differences were observed in other genes (Figure 3B). Therefore, we finally chose PIK3CA (encoding PI3K) gene for further investigation.

### MiR-29a targeted PIK3CA and inhibited atherosclerotic plaque formation *in vivo*

**Figure 4:**
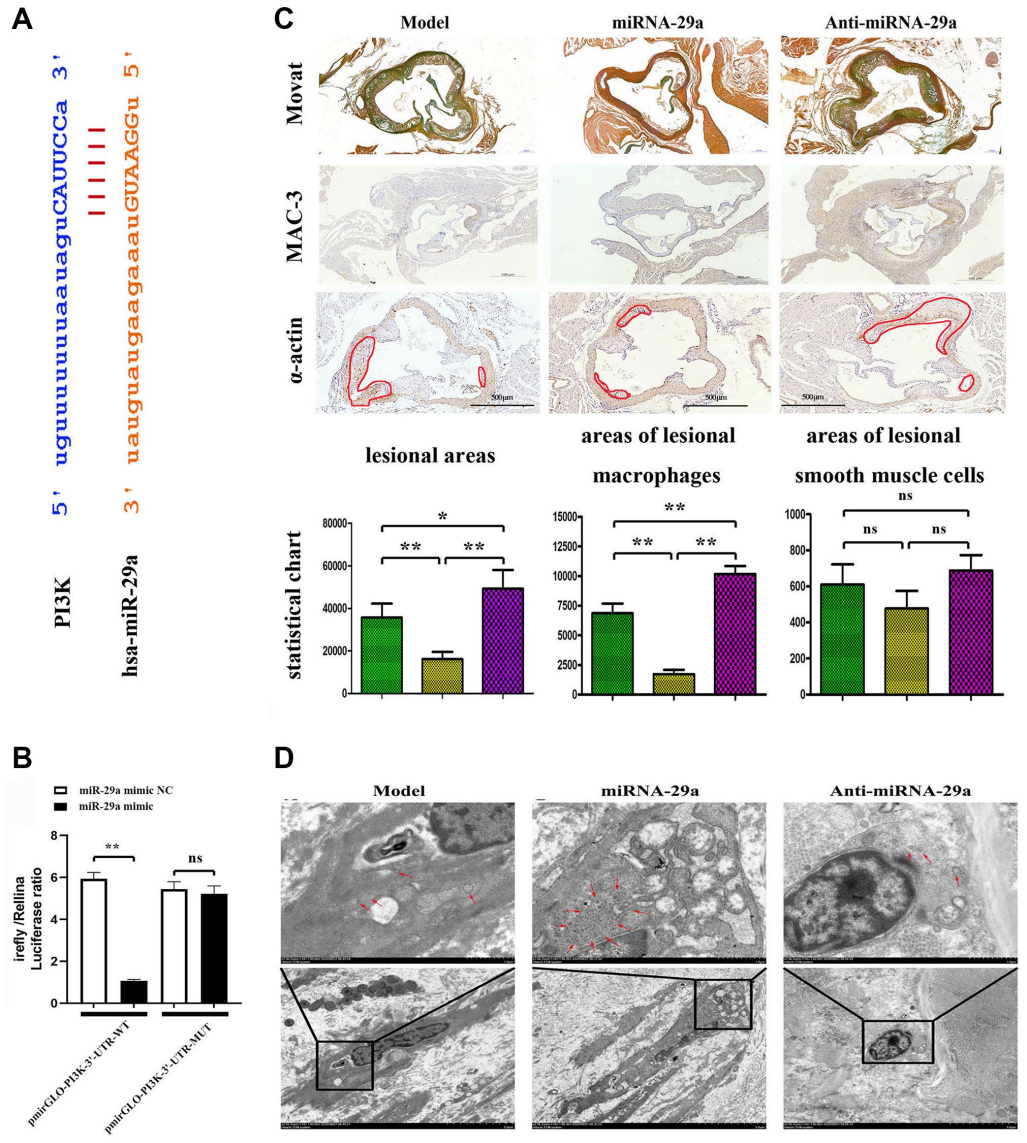
**MiR-29a targets PIK3CA and inhibits atherosclerotic plaque formation *in vivo*.** (**A**) MiR-29a binds to the PIK3CA gene. (**B**) The luciferase activity of pmirGLO reporter gene is reduced after co-transfection with pmirGLO-PI3K-3'-UTR-WT and hsa-miR-29a mimic, but it shows no statistically significant difference after co-transfection with pmirGLO-PI3K-3'-UTR-MUT and hsa-miR-29a mimic. (**C**) MOVAT staining and immunohistochemical staining of MAC-3 and α-SMA are performed to investigate the atherosclerotic lesion area in mice. (**D**) TEM is adopted to investigate the macrophage morphological changes. Data are expressed as the mean ± SD. *P* < 0.05.

The online biological prediction tool showed that miR-29a could bind to the PIK3CA gene (Figure 4A). The luciferase activity of pmirGLO reporter gene was reduced after co-transfection with pmirGLO-PI3K-3′-UTR-WT and hsa-miR-29a mimic, but it showed no statistically significant difference after co-transfection with pmirGLO-PI3K-3′-UTR-MUT and hsa-miR-29a mimic (Figure 4B). *In vivo*, the effect of miR-29a on AS was examined using miR-29a mimic/ApoE^−/−^mice and miR-29a NC/ApoE^−/−^mice. All mice fed with a high-fat diet suffered from AS. The atherosclerotic lesion area in mice, as determined by MOVAT staining and immunohistochemical staining of MAC-3 and α-SMA, was significantly decreased in miR-29a group, but significantly increased by anti-miR-29a administration (Figure 4C). Our quantitative analysis revealed that the lesion area in anti-miR-29a group was reduced compared with that in model group. (*P* < 0.05, Figure 4C). Moreover, TEM results revealed that model group exhibited reduced matrix density and noticeable morphological changes, which could be enhanced by miR-29a administration (Figure 4D). These results indicated that miR-29a inhibits atherosclerotic plaque formation and progression in AS mice.

### MiR-29a increased the expressions of IL-10, Mrc1 and Arginase-1 and decreased the expressions of IFN-γ, IL-1β and iNOS *in vivo*

**Figure 5:**
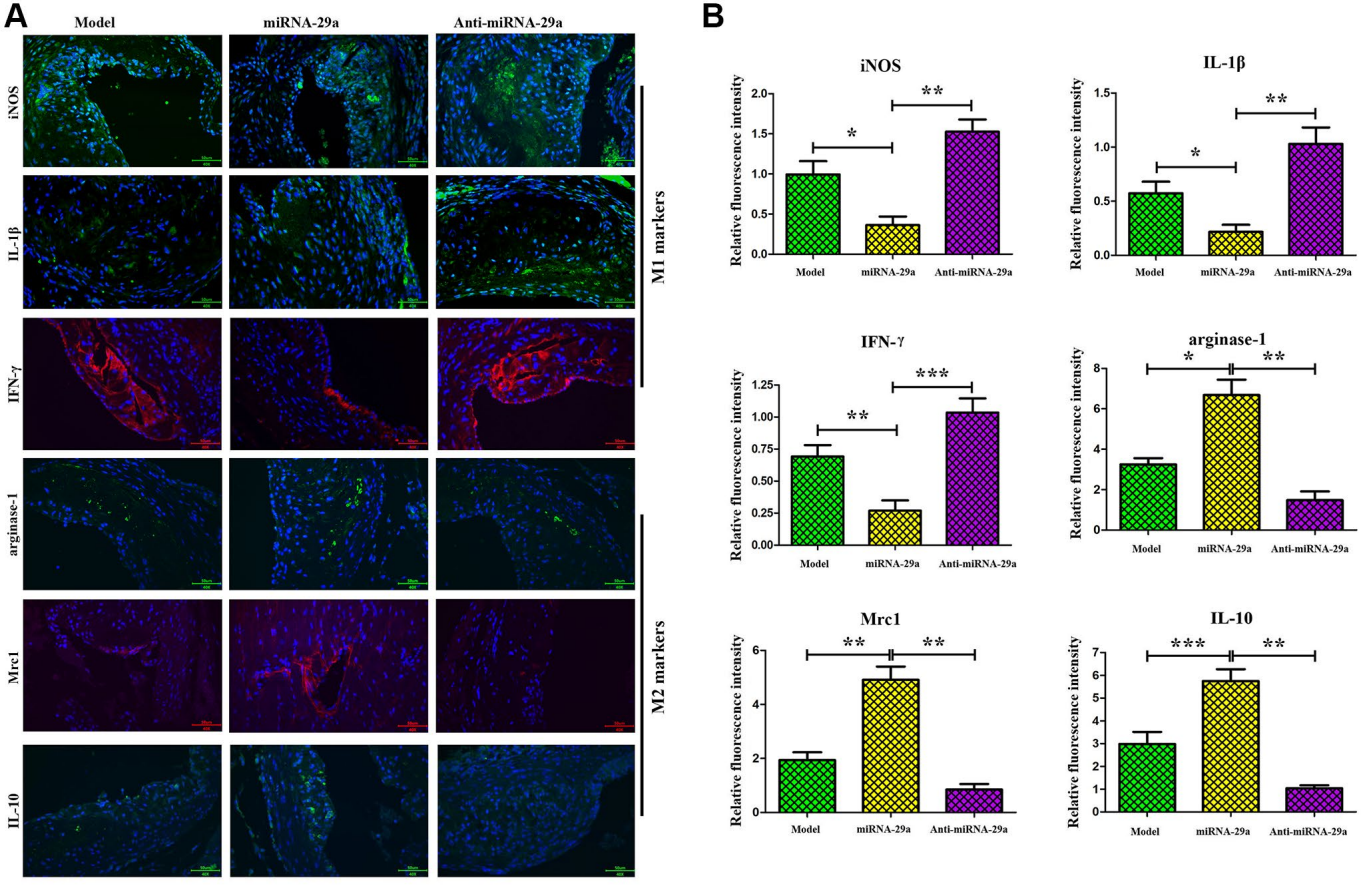
**MiR-29a increases the expressions of M2-like macrophages markers (Arginase-1, Mrc-1 and IL-10) and decreases the expressions of M1-like macrophages markers (iNOS, IL-1β and IFN-γ) *in vivo*.** (**A**) The expressions of these proteins are detected by immunofluorescence assay. (**B**) The relative fluorescence intensities of Arginase-1, Mrc-1, IL-10, iNOS, IL-1β and IFN-γ. Data are expressed as the mean ± SD. *P* < 0.05. Model group vs. miR-29a group; miR-29a group vs. anti-miR-29a group.

IFN-γ and IL-10 are key inflammatory factors that play essential roles in the progression of AS [17, 18]. Further investigation of macrophage phenotype, characterized as M1 pro-inflammatory macrophage, was performed using the markers IFN-γ, IL-1β and iNOS and M2-like macrophage markers, IL-10, Mrc1 and Arginase-1. The alterations of IL-10 and IFN-γ expressions in the atherosclerotic lesions were identified by immunofluorescence assay. The results showed that the expressions of IL-10, Mrc1 and Arginase-1 were increased, whereas those of IFN-γ, IL-1β and iNOS were decreased in miR-29a group (Figure 5A), which were identical to the results of the quantitative analysis (Figure 5B, *P* < 0.05; model group vs. miR-29a group; miR-29a group vs. anti-miR-29a group). These findings indicated that miR-29a can induce anti-inflammatory M2-like macrophages and inhibit pro-inflammatory M1-like macrophages in AS mice.

### MiR-29a overexpression increased autophagy and suppressed the PI3K/AKT/mTOR pathway

**Figure 6:**
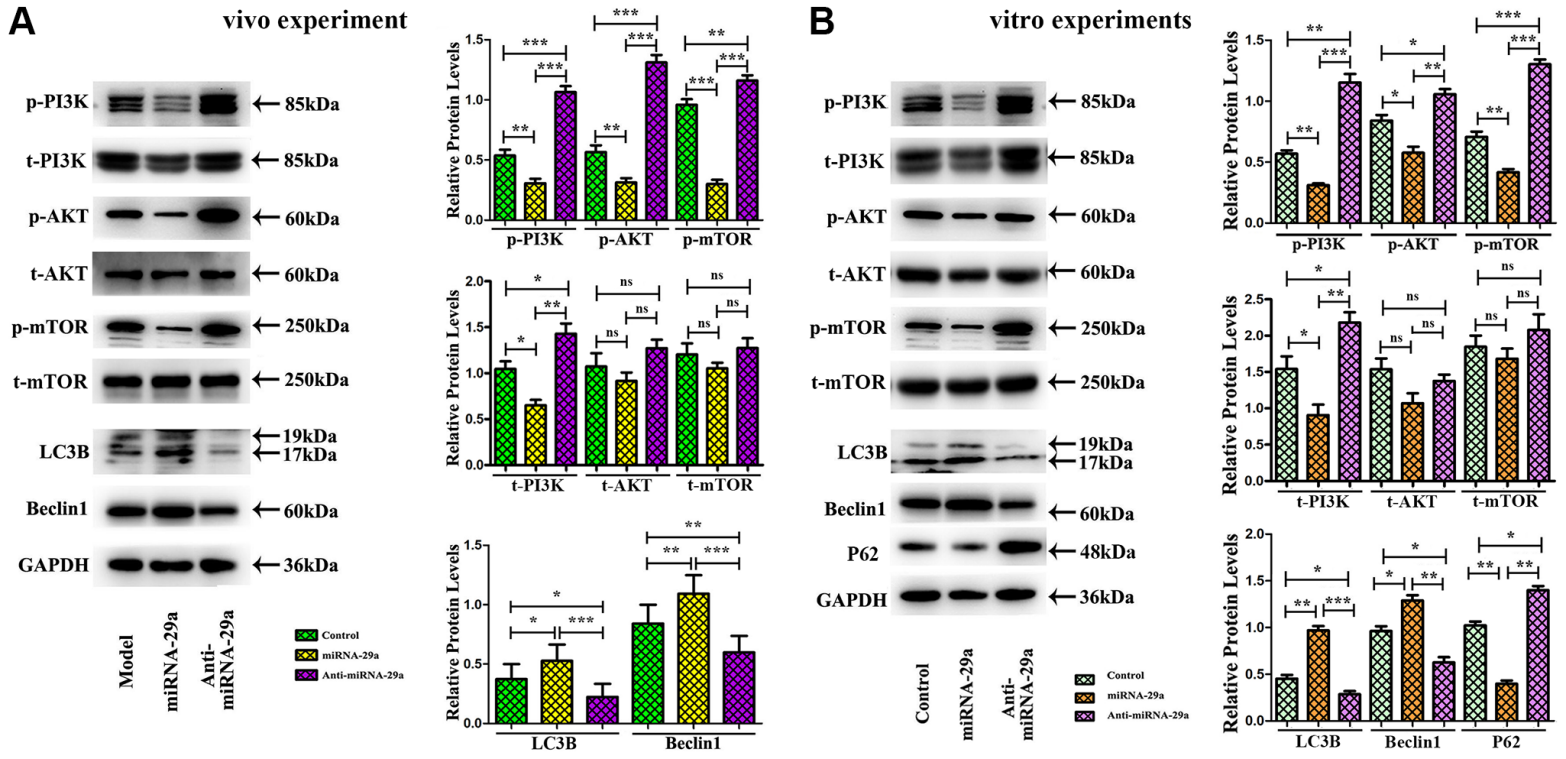
**MiR-29a overexpression inhibits AS by increasing autophagy and suppressing PI3K/AKT/mTOR pathway.** (**A**) Western blotting reveals that the protein expressions of p-PI3K, total-PI3K, p-AKT, and p-mTOR are decreased, while those of Beclin 1 and LC3II are increased in miR-29a group *in vivo*. (**B**) Western blotting reveals that the protein expressions of p-PI3K, total-PI3K, p-AKT, p-mTOR and P62 are decreased, while those of Beclin 1 and LC3II are increased in miR-29a group *in vitro*.

To further investigate the effect of miR-29a on the PI310.1042/BSR20200353K/AKT/mTOR signaling pathway, we performed a Western blotting analysis. *In vivo*, the protein expressions of p-PI3K, total-PI3K (t-PI3K), p-AKT, and p-mTOR were decreased in miR-29a group, while the expression levels of autophagy-related proteins, Beclin 1 and LC3II, were increased in miR-29a group, suggesting that miR-29a overexpression can enhance autophagy and inhibit the PI3K/AKT/mTOR pathway in AS mice (Figure 6A).

In *in vitro* experiments, RAW264.7 cells were transfected with miR-29a NC, miR-29a mimic, or anti-miR-29a mimic. According to Western blotting results, the expression levels of p-PI3K, t-PI3K, p-AKT, p-mTOR and P62 (the marker for autophagy flux) were reduced, whereas those of Beclin 1 and LC3II were enhanced by miR-29a mimic administration in miR-29a mimic group (Figure 6B). As shown in Figure 6A, 6B, the quantitative analysis of relative protein levels showed a similar tendency as the Western blotting analysis (*P* < 0.05). It can be concluded that miR-29a inhibits the PI3K/AKT/mTOR pathway to elevate the autophagy level in the atherosclerotic environment.

### MiR-29a elevation enhanced macrophage autophagy

**Figure 7:**
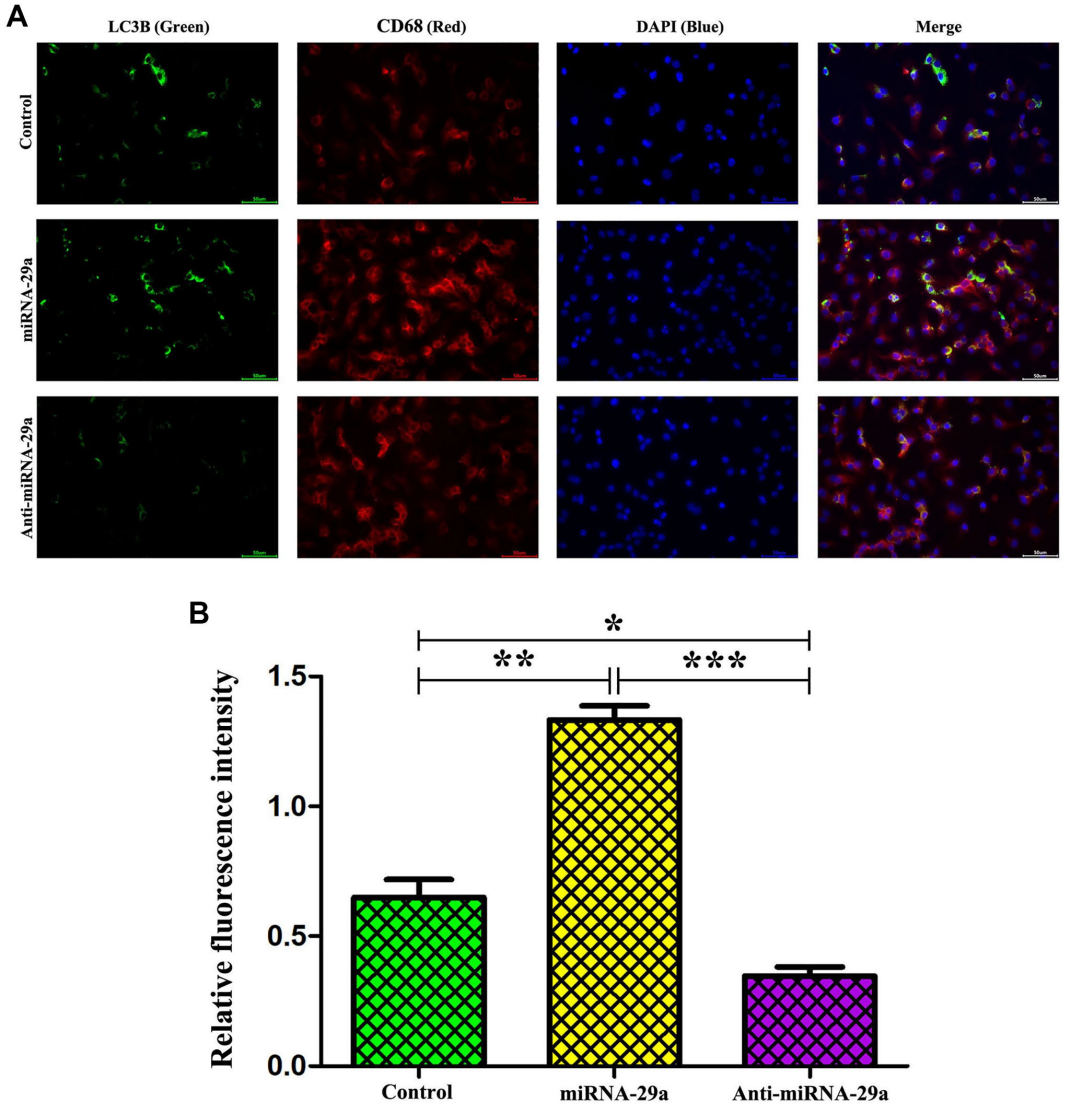
**MiR-29a elevation enhances macrophage autophagy.** (**A**) Administration of miR-29a significantly up-regulates the expression of LC3II in macrophages. (**B**) The quantitative analysis of relative fluorescence intensity reveals that the expression of LC3II is increased, but it was significantly decreased by anti-miR-29a in miR-29a group. Data are expressed as the mean ± SD. *P* < 0.05. Model group vs. miR-29a group; miR-29a group vs. anti-miR-29a group.

To further determine whether macrophages specifically expressed LC3II, an autophagosome marker, immunofluorescence staining was performed to evaluate macrophages and LC3II *in vitro*. The results showed that the administration of miR-29a significantly up-regulated the expression of LC3II in macrophages, and LC3II could be co-localized primarily to macrophages (Figure 7A). Furthermore, the quantitative analysis of relative fluorescence intensity revealed that the expression of LC3II was increased in miR-29a group, but decreased by anti-miR-29a (Figure 7B, all *P* < 0.05). These results implied that miR-29a elevation enhances macrophage autophagy.

## DISCUSSION

AS is a chronic inflammatory disease characterized by lipid metabolism disorder, calcareous deposition, vascular sclerosis, and vascular stenosis [19]. It has been confirmed that AS is the leading cause of many severe cardiovascular and cerebrovascular diseases [20], so it is urgently needed to find effective prevention and treatment measures for the disease. As previously described, miRNAs have been proven as significant regulators of the physiological homeostasis of the cardiovascular system [21]. The present study aims to investigate the role of miR-29a in the development of AS. The results of *in vivo* and *in vitro* experiments revealed that the upregulation of miR-29a expression inhibited AS via downregulating the PI3K/AKT/mTOR signaling pathway to enhance the autophagy.

The formation and progression of AS are reversible processes involving endothelial cell injury, lipid disorder, hemodynamic abnormality, and autophagy of macrophages, among which the autophagy of macrophages plays an essential role [22]. Macrophages can not only reduce the accumulation of foam cells and inhibit the formation and development of plaques in the early stage of AS, but also reduce plaque inflammation and stabilize the plaques in the middle and late stages of the disease [23].

In the present study, firstly, the gene expression profiles of GSE137578, GSE132651, GSE113969 and GSE97210 were selected from the Gene Expression Omnibus (GEO) database, which were used as the samples to investigate the cellular/molecular mechanisms of AS. Secondly, the gene expression levels of the 4 GEO datasets were standardized by means of quartile division and the data before and after standardization were obtained. Thirdly, volcano maps of the top 100 DEGs in these datasets were plotted. Finally, the integrated DEGs of each dataset were obtained through the Venn diagram analysis. In addition, GO and KEGG enrichment analyses of the overlapping DEGs revealed that autophagy was an enriched pathway involved in AS. Using Cytoscape software and STRING database, we constructed the PPI network on the overlapping DEGs, and 6 genes with the highest scores were obtained as hub genes. Compared with that in control group, the expression of PIK3CA was higher in AS group, but no statistically significant differences were observed in other genes. Thus, we finally chose PIK3CA gene and the autophagy pathway for further investigation.

Autophagy is a key catabolic cycle pathway that involves the degradation of different substrates, such as misfolded proteins, lipids, or damaged organelles, which contributes to cell recovery and plays an important role in the development of AS [24]. When the body is stimulated by AS inflammation, hunger, or oxidative stress, AS-related vascular cells will start autophagy, showing a correlation between AS and autophagy [25]. The early initiation of autophagy can protect vascular cells and delay the formation of AS to a certain extent, while excessive autophagy can lead to the apoptosis of vascular cells and accelerate the rupture of atherosclerotic plaques [26]. Vascular autophagy exists in atherosclerotic plaques, indicating that autophagy participates in and regulates a series of processes of AS occurrence [27, 28]. In addition, previous studies have shown that the properly activated autophagy pathway promotes the M1-M2 transition of advanced atherosclerotic plaques, alleviates inflammatory responses, and improves the occurrence and development of atherosclerotic plaques [29].

Inflammatory mechanism is a relatively classic promoter in the pathogenesis of AS, and macrophages, as inflammatory cells, play an important role in the inflammatory response of AS [30]. Research has shown that macrophages infiltrate lesions and participate in the progression of the plaques, in which different types of polarization of macrophages in the atherosclerotic inflammation are crucial. Besides, macrophages are involved in the entire development course of AS, and the quantity, phenotype and migration of macrophages are closely related to the fate of atherosclerotic plaques. For example, the subtypes of polarization are considered as "keys" to the development, and they affect plaque stability and AS outcome [31]. Activated macrophages can be divided into pro-inflammatory (M1) and anti-inflammatory (M2) types according to their characteristic stimulation. Classically activated M1-like macrophages are mainly present in unstable plaques of symptomatic patients, mainly in the areas prone to rupture, and they promote the production of pro-atherogenic inflammatory mediators and tissue-degrading enzymes, such as matrix metalloproteinases, leading to persistent inflammation and plaque vulnerability [32, 33]. In contrast, M2-like macrophages, primarily produced by IL-4 and IL-13 and expressed primarily in asymptomatic plaques, are resistant to cholesterol load, exhibit a greater capacity to store esterified cholesterol, and promote fibrous cap formation by promoting collagen production [34]. When the body is stimulated by AS inflammation, hunger or oxidative stress, vascular cells associated with AS will start autophagy, showing a relevance to autophagy [27]. The early initiation of autophagy can protect vascular cells and delay the formation of AS to a certain extent, while excessive autophagy can lead to the apoptosis of vascular cells and accelerate the rupture of atherosclerotic plaques [26]. The phenomenon of vascular autophagy exists in atherosclerotic plaques, indicating that autophagy participates in and regulates a series of processes of AS occurrence [27, 28]. Recent studies have indicated that autophagy also plays an important role in regulating the functional polarization of macrophages. The PI3K/AKT/mTOR autophagy signaling pathway serves as the master mediator for the cross talk of macrophage’ functional polarization and autophagy in AS [35, 36]. The M2 polarization of macrophages containing constitutively activated mTORC1 is defective both *in vivo* and *in vitro* [24, 25]. In addition, previous studies have shown that the appropriate activation of autophagy pathway promotes the M1-M2 transition of AS, alleviates inflammatory responses, and improves the occurrence and development of atherosclerotic plaques [29]. Additionally, an online biological prediction tool indicated that miR-29a could bind to the PIK3CA (encoding PI3K) gene. Thus, it was confirmed that the miR-29a inhibited the PI3K/AKT/mTOR signals, amplified the early or stable autophagy progression of atherosclerotic plaques, and suppressed the inflammatory responses as well as lesional expansion. Moreover, we found the decreased critical inflammatory factors consistent with anti-inflammatory macrophage phenotype [17, 18], indicating that miR-29a can induce anti-inflammatory M2-like macrophages and inhibit pro-inflammatory M1-like macrophages in AS mice.

Previous studies have indicated that the PI3K/AKT/mTOR signaling pathway plays a critical regulatory role in autophagy [11]. We investigated the specific effects of miR-29a on autophagy and PI3K/AKT/mTOR signaling pathway. *In vivo*, the protein expressions of p-PI3K, p-AKT, p-mTOR and P62 (the marker for autophagy flux) declined, but those of autophagy-related proteins, Beclin 1 and LC3II rose in miR-29a group, suggesting that miR-29a overexpression enhances autophagy and inhibits the PI3K/AKT/mTOR pathway in AS mice. The above results were identified by *in vivo* and *in vitro* experiments. In conclusion, the present study evidences that miR-29a elevation can induce the increase of autophagy by down-regulating the PI3K/AKT/mTOR pathway in the progression of AS, indicating a translational value of miR-29a as a novel therapeutic strategy for AS.
